# Stem cells in aged mammalian ovaries

**DOI:** 10.18632/aging.100117

**Published:** 2010-01-26

**Authors:** Irma Virant-Klun, Thomas Skutella

**Affiliations:** ^1^ Department of Obstetrics and Gynaecology, University Medical Center Ljubljana, Ljubljana, Slovenia; ^2^ Department of Experimental Embryology and Center for Regenerative Biology and Medicine, University of Tübingen, Tübingen, Germany

**Keywords:** aging, cancer, ovary, ovarian surface epithelium, stem cells

The ovary undergoes several changes after the menopause. The main characteristics of the postmenopausal ovary are a loss of follicles and several changes as a consequence of apoptotic processes. Signs of atrophy and fibrosis are evident. Primordial follicles are usually absent in postmenopause, whereas corpora atretica, hemorrhagica and albicantia, scar tissue, and simple follicular cysts are common.

## Ovarian surface epithelium during the embryonic and reproductive periods of life

Ovarian surface epithelium (OSE) is an important structure of the human ovary and is involved in both reproductive function and ovarian tumor formation. Primordial germ cells (PGCs) in embryonic ovaries are of extraovarian origin, but those developing during the fetal period are derived from the OSE. PGCs in the fetal ovary express most, but not all of the markers associated with pluripotent cells [1] and can develop into pluripotent stem cells such as embryonic germ cells (EGCs) and embryonic carcinoma cells (ECCs). With the support of the immune system cells, secondary germ cells and primitive granulosa cells arise from the OSE stem cells in the fetal gonads. Additionally, human neonatal thecal stem cells have already been isolated, characterized, and differentiated *in vitro* and *in vivo*[2]. Because of its physiological role during the fetal  period of life, the OSE layer has also been termed *"germinal epithelium." *During the adult, reproductive period of life, OSE is mainly involved in the physiological process of ovulation. Ovulation induces cyclic rupture and regenerative repair of the ovarian coelomic epithelium. This process of repeated disruption and repair accompanied by the complex remodeling reflects a somatic stem/progenitor cell-mediated process in the mammalian ovaries; a label-retaining cell population in the coelomic epithelium of the adult mouse ovary has already been identified as possible somatic stem/progenitor cells [3].

## OSE and stem cells in postmenopausal women

The OSE layer gradually flattens, but is always present, even in menopausal females of advanced age [4]. Apoptotic and necrotic cells frequently appear within the OSE layer.

It seems that human OSE stem cells retain the characteristics of embryonic stem cells. Based on previous experience [5], Virant-Klun and her coworkers [6, 7] scraped the OSE in women with no naturally occurring oocytes and follicles: postmenopausal women and young women with premature ovarian failure  (POF).

**Figure 1.**
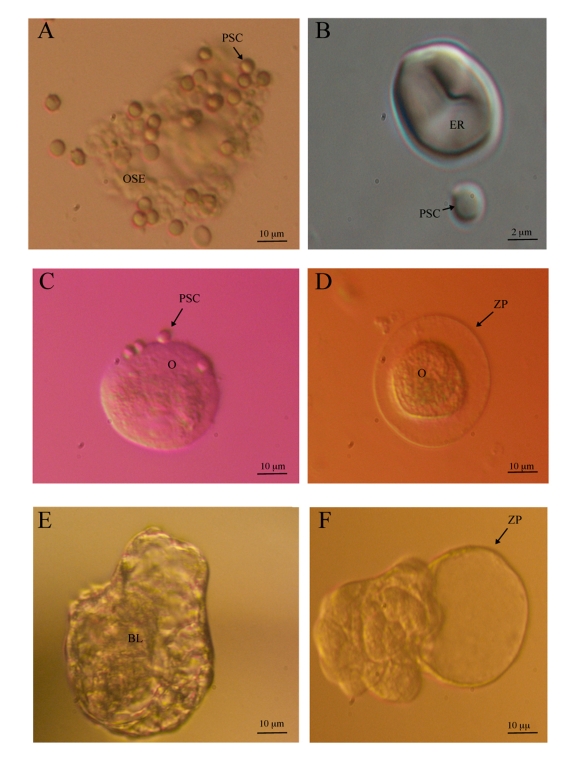
**Ovarian surface epithelium stem cells and *in vitro* developed oocyte-like cells in postmenopausal women.** (**A**) Putative stem cells proliferating and growing in the cluster of scraped OSE cells (inverted microscope, Hoffman illumination, magnification 400x). (**B**) Putative stem cell and erythrocyte (inverted microscope equipped with Nikon Digital Sight DS-Ri1 camera, DIC-Nomarski illumination, magnification 6000x).  (**C**) Oocyte-like cell without zona pellucida with attached putative stem cells possibly acting as granulosa cells (inverted microscope, Hoffman illumination, magnification 200x). (**D**) Oocyte-like cell with zona pellucida-like structure attached to the dish bottom (inverted microscope, Hoffman illumination, magnification 200x). (**E**) Blastocyst-like structure with blastocoel-like cavity at the beginning of **"**hatching**"**(inverted microscope, Hoffman illumination, magnification 100x). (**F**) Blastocyst-like structure after **"**hatching**"** (inverted microscope, Hoffman illumination, magnification 100x) (images: *University Medical Centre Ljubljana, 2009*).

In the scraped population of cells, which consisted primarily of epithelial cells, they found small, round, yellow-colored cells with a diameter of 2 to 4 μm and small, bubble-like structures; the cells did not respond to blood or immune-system-related cells (Figure 1A and 1B). Similar cells were also found histologically in the ovarian sections of patients. These cells were immunomagnetically isolated from the remaining population of cells and expressed a number of transcription factors and surface antigens characteristic of pluripotent embryonic stem cells. In the presence of heterologous follicular fluid obtained from the institute's *in vitro* fertilization program, these cells developed into round oocyte-like cells (Figure 1C and 1D) expressing a number of oocyte-specific genes. Some of these cells spontaneously developed into blastocyst-like structures (Figure 1E and 1F) which could be a kind of parthenogenetic embryos [7]. The investigators concluded that they had discovered small embryonic-like stem cells comparable to embryonic-like stem cells found in other adult human tissues and organs [8,9,10,11]. Although these small embryonic-like stem cells remain to be further characterized, they might persist in adult tissues from the embryonic period of life and play a role in rejuvenation and longevity. There have also been reports of the *in vitro* development of embryonic stem cells into oocyte-like cells in the mouse [12,13,14,15].

Similar types of stem cells were found in the mouse [16]. Neonatal and adult mouse germline stem cell lines were established after immunomagnetic isolation. These lines expressed a normal karyotype and high telomerase activity and could be cultured for several months.

Recently, the Tilly's group performed important experiments on the adult mouse ovary. They found that the germline-specific meiosis-commitment genes *Stimulated by retinoic acid 8* (*Stra-8*) and *Deleted in azoospermia-like* (*Dazl*) are highly expressed in aged mouse ovaries with complete oocyte depletion [17]. In the OSE layer of aged mouse ovaries, they found a rare population of premeiotic germ cells which expressed the *Stra 8* gene and failed to develop further. These cells retained the capacity to develop into oocytes when transplanted and exposed to a young host environment. Premeiotic germ cells apparently persist in aged atrophic mouse ovaries but are blocked in their ability to undergo meiosis and transition into oocytes.

## Potential role of OSE stem cells

In spite of the persistent dogma that the number of follicles and oocytes in the mammalian ovary is set at birth and depleted over the course of life, a role of ovarian stem cells in *de novo* folliculogenesis and oogenesis in the adult ovary cannot be ruled out. It is difficult to prove *de novo* oogenesis/folliculogenesis in humans, because there are strict limitations on the methodologies allowed in *in vivo* studies. Tilly's group was the first to confirm this in the mouse model. They demonstrated cells expressing the meiotic protein SCP3 in juvenile and adult mouse ovaries after the previous elimination of the primordial follicle reserve with the cell toxin busulfan [18]. They found that wild-type ovaries grafted into transgenic female mice with green fluorescent protein (GFP) expression become infiltrated with GFP-positive germ cells that form follicles [18]. Furthermore, they confirmed the formation of immature oocytes after bone marrow transplantation into the mice with previously induced premature ovarian failure [19]. Analogously, a bone marrow mesenchymal stem cell transplantation improved the ovarian function and structure in rats with chemotherapy-induced ovarian damage [20]. Zou and co-workers [16] infected mouse germline stem cells with GFP virus and transplanted them into ovaries of infertile mice. The transplanted cells underwent oogenesis and the mice produced offspring that had the GFP transgene.

Two main scientific facts support the idea of *de novo* oogenesis and folliculogenesis in the adult human ovary: firstly, the presence of stem cells in the human adult OSE [6,7] and in the human mature vesicular (Graafian) follicles [21] as well as extrafolliculary, as confirmed in the mouse adult ovary [22], and secondly, the phenomenon of epithelial-mesenchymal transitions. It has already been confirmed that the OSE shows characteristics of both mesenchymal and epithelial cells and that under mostly unknown conditions epithelial cells can be transformed into mesenchymal cells [23].

## OSE stem cells and ovarian cancer

Most ovarian cancers arise from the OSE - mesothelial surface lining of the ovaries or from invaginations of this lining into the superficial ovarian cortex which form cortical inclusion cysts. These cysts are thought to be precursor lesions of ovarian carcinomas. Epithelial-mesenchymal transition, a transcriptional program inducing maintenance of the mesenchymal phenotype, plays a role in tumor progression and metastasis [23]. Aggressive epithelial ovarian cancer (EOC) is genetically and epigenetically distinct from the normal OSE and early neoplasia. Co-expression of epithelial and mesenchymal markers in EOC suggests an involvement of epithelial-mesenchymal transition in cancer initiation and progression [24]. Gene expression profiling supports the hypothesis that human ovarian surface epithelia are multipotent and capable of serving as ovarian cancer-initiating cells [25]. They express certain transcription factors characteristic of embryonic stem cells [26]. Indeed, there is more and more evidence of and acceptance of the concept of a stem cell origin of ovarian tumors [27,28,29,30]. Such a stem cell origin might explain the high resistance of ovarian tumors to chemo- and radiotherapy and their lethality.

Stem cells present in the OSE layer of postmenopausal women can no longer be involved in reproductive function (*de novo* oogenesis/folliculogenesis) due to different natural blockades in the ovary, but they can be involved in the formation of ovarian cancer. Because they are accumulated in the OSE layer, they can quickly develop into ovarian tumors. This might be the reason for the clinical experience of a higher incidence of epithelial ovarian cancer in older, postmenopausal women [31] and for the connection between ovarian epithelial cancer and the depletion of follicles in the human ovary [32].

## References

[1] Kerr CL, Hill CM, Blumenthal PD, Gearhart JD (2008). Expression of pluripotent stem cell markers in the human fetal ovary. Hum Reprod.

[2] Honda A, Hirose M, Hara K, Matoba S, Inoue K, Miki H, Hiura H, Kanatsu-Shinohara M, Kanai Y, Kono T, Shinohara T, Ogura A (2007). Isolation, characterization, and in vitro and in vivo differentiation of putative thecal stem cells. Proc Natl Acad Sci U S A.

[3] Szotek PP, Chang HL, Brennand K, Fujino A, Pieretti-Vanmarcke R, Lo Celso C, Dombkowski D, Preffer F, Cohen KS, Teixeira J, Donahoe PK (2008). Normal ovarian surface epithelial label-retaining cells exhibit stem/progenitor cell characteristics. Proc Natl Acad Sci U S A.

[4] Motta PM, Heyn R, Makabe S (2002). Three-dimensional microanatomical dynamics of the ovary in postreproductive aged women. Fertil Steril.

[5] Bukovsky A, Svetlikova M, Caudle MR (2005). Oogenesis in cultures derived from adult human ovaries. Reprod Biol Endocrinol.

[6] Virant-Klun I, Zech N, Rozman P, Vogler A, Cvjeticanin B, Klemenc P, Malicev E, Meden-Vrtovec H (2008). Putative stem cells with an embryonic character isolated from the ovarian surface epithelium of women with no naturally present follicles and oocytes. Differentiation.

[7] Virant-Klun I, Rozman P, Cvjeticanin B, Vrtacnik-Bokal E, Novakovic S, Rülicke T, Dovc P, Meden-Vrtovec H (2009). Parthenogenetic embryo-like structures in the human ovarian surface epithelium cell culture in postmenopausal women with no naturally present follicles and oocytes. Stem Cells Dev.

[8] Kucia M, Wysoczynski M, Ratajczak J, Ratajczak MZ (2008). Identification of very small embryonic like (VSEL) stem cells in bone marrow. Cell Tissue Res.

[9] Ratajczak MZ, Zuba-Surma EK, Shin DM, Ratajczak J, Kucia M (2008). Very small embryonic-like (VSEL) stem cells in adult organs and their potential role in rejuvenation of tissues and longevity. Exp Gerontol.

[10] Ratajczak MZ, Kucia M, Ratajczak J, Zuba-Surma EK (2009). A multi-instrumental approach to identify and purify very small embryonic like stem cells (VSELs) from adult tissues. Micron.

[11] Zuba-Surma EK, Kucia M, Ratajczak J, Ratajczak MZ (2009). "Small stem cells" in adult tissues: very small embryonic-like stem cells stand up. Cytometry.

[12] Hübner K, Fuhrmann G, Christenson LK, Kehler J, Reinbold R, De La Fuente R, Wood J, Strauss JF 3rd, Boiani M, Schöler HR (2003). Derivation of oocytes from mouse embryonic stem cells. Science.

[13] Lacham-Kaplan O, Chy H, Trounson A (2006). Testicular cell conditioned medium supports differentiation of embryonic stem cells into ovarian structures containing oocytes. Stem Cells.

[14] Novak I, Lightfoot DA, Wang H, Eriksson A, Mahdy E, Höög C (2006). Mouse embryonic stem cells form follicle-like ovarian structures but do not progress through meiosis. Stem Cells.

[15] Qing T, Shi Y, Qin H, Ye X, Wei W, Liu H, Ding M, Deng H (2007). Induction of oocyte-like cells from mouse embryonic stem cells by co-culture with ovarian granulosa cells. Differentiation.

[16] Zou K, Yuan Z, Yang Z, Luo H, Sun K, Zhou L, Xiang J, Shi L, Yu Q, Zhang Y, Hou R, Wu J (2009). Production of offspring from a germline stem cell line derived from neonatal ovaries. Nat Cell Biol.

[17] Niikura Y, Niikura T, Tilly JL (2009). Aged mouse ovaries possess rare premeiotic germ cells that can generate oocytes following transplantation into a young host environment. Aging.

[18] Johnson J, Canning J, Kaneko T, Pru JK, Tilly JL (2004). Germline stem cells and follicular renewal in the postnatal mammalian ovary. Nature.

[19] Lee HJ, Selesniemi K, Niikura Y, Niikura T, Klein R, Dombkowski DM, Tilly JL (2007). Bone marrow transplantation generates immature oocytes and rescues long-term fertility in a preclinical mouse model of chemotherapy-induced premature ovarian failure. J Clin Oncol.

[20] Fu X, He Y, Xie C, Liu W (2008). Bone marrow mesenchymal stem cell transplantation improves ovarian function and structure in rats with chemotherapy-induced ovarian damage. Cytotherapy.

[21] Kossowska-Tomaszczuk K, De Geyter C, De Geyter M, Martin I, Holzgreve W, Scherberich A, Zhang H (2009). The multipotency of luteinizing granulosa cells collected from mature ovarian follicles. Stem Cells.

[22] Zhang D, Fouad H, Zoma WD, Salama SA, Wentz MJ, Al-Hendy A (2008). Expression of stem and germ cell markers within nonfollicle structures in adult mouse ovary. Reprod Sci.

[23] Okamoto S, Okamoto A, Nikaido T, Saito M, Takao M, Yanaihara N, Takakura S, Ochiai K, Tanaka T (2009). Mesenchymal to epithelial transition in the human ovarian surface epithelium focusing on inclusion cysts. Oncol Rep.

[24] Berry NB, Bapat SA (2008). Ovarian cancer plasticity and epigenomics in the acquisition of a stem-like phenotype. J Ovarian Res.

[25] Bowen NJ, Walker LD, Matyunina LV, Logani S, Totten KA, Benigno BB, McDonald JF (2009). Gene expression profiling supports the hypothesis that human ovarian surface epithelia are multipotent and capable of serving as ovarian cancer initiating cells. BMC Med Genomics.

[26] Lu L, Katsaros D, Shaverdashvili K, Qian B, Wu Y, de la Longrais IA, Preti M, Menato G, Yu H (2009). Pluripotent factor lin-28 and its homologue lin-28b in epithelial ovarian cancer and their associations with disease outcomes and expression of let-7a and IGF-II. Eur J Cancer.

[27] Pan Y, Huang X (2008). Epithelial ovarian cancer stem cells-a review. Int J Clin Exp Med.

[28] Ponnusamy MP, Batra SK (2008). Ovarian cancer: emerging concept on cancer stem cells. J Ovarian Res.

[29] Gauthaman K, Fong CY, Bongso A (2009). Statins, stem cells, and cancer. J Cell Biochem.

[30] Fong MY, Kakar SS (2010). The role of cancer stem cells and the side population in epithelial ovarian cancer. Histol Histopathol.

[31] Mørch LS, Løkkegaard E, Andreasen AH, Krüger-Kjaer S, Lidegaard O (2009). Hormone therapy and ovarian cancer. JAMA.

[32] Smith ER, Xu XX (2008). Ovarian ageing, follicle depletion, and cancer: a hypothesis for the aetiology of epithelial ovarian cancer involving follicle depletion. Lancet Oncol.

